# Supplementary material for: Nanoscale feedback control of six degrees of freedom of a near-sphere
Source: Nat Commun. 2023 Dec 1;14:7943. doi: 10.1038/s41467-023-43745-7 (PMC10692201; doi:10.1038/s41467-023-43745-7)
Supplement: Supplementary file 1 — Supplementary Information [file 41467_2023_43745_MOESM1_ESM.pdf]

Supplementary Information

Supplementary Figure and Supplementary Table

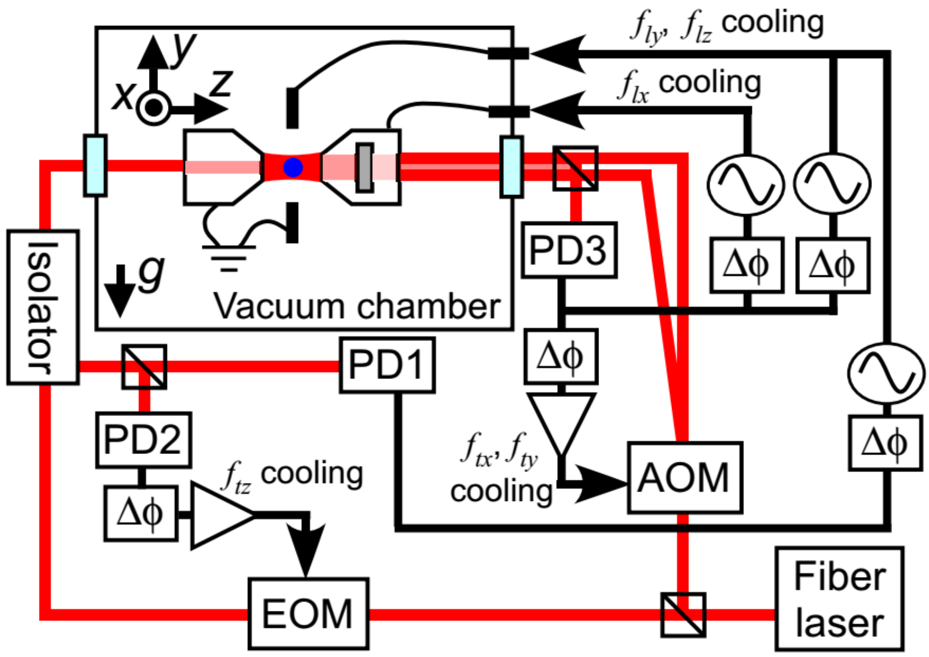

Supplementary Figure 1 Complete schematic of our experimental setup. A nearly spherical silica nanoparticle is trapped in an optical lattice. Translational motions and librational motions are observed via three photodetectors. The translational motions are feedback-cooled by modulating optical gradients. The librational motions are feedback-cooled by applying electric fields including three frequencies that are phase-locked to each librational motion. The electrodes for cooling the motions at f_{ly} and f_{lz} are tilted by 45° with respect to the x axis.

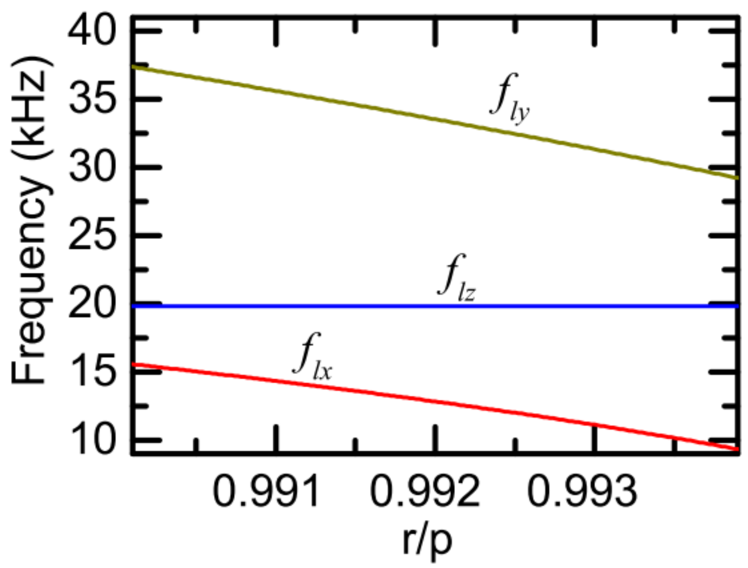

Supplementary Figure 2 Calculated librational frequencies with respect to *r/p*. Three librational frequencies are calculated with eqs.(3). q/p is set to 0.9961. The observed librational frequencies are reproduced within 0.7 % when r/p is 0.9914.

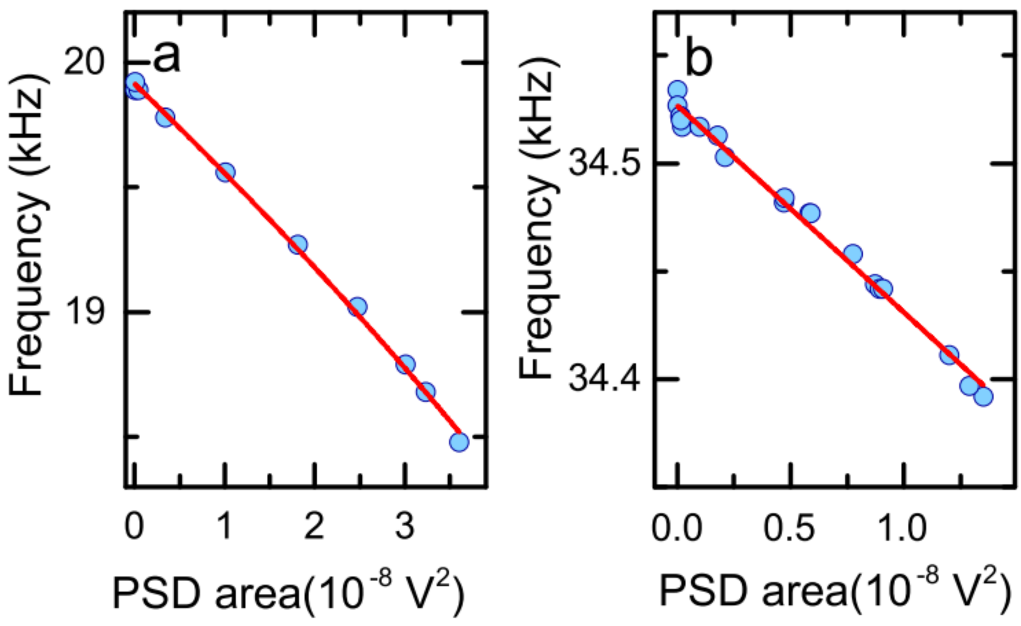

Supplementary Figure 3　Measured librational frequencies with respect to the area of the PSD.　**a,** For f_{lz}. **b,** For f_{ly}. The solid lines are fits with eq.(1). The variations of librational frequencies due to the nonlinearity of the angular potential are used for the thermometry of librational motions in each direction.

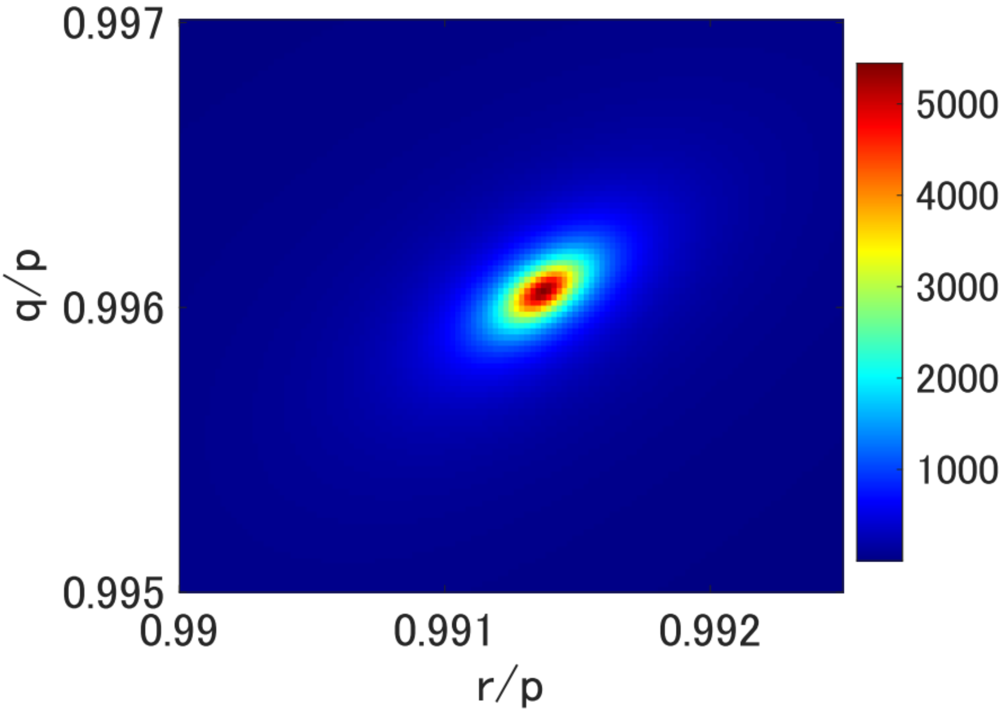

Supplementary Figure 4　Two dimensional plot of $1/\delta$ as functions of the radii *q/p*, *r/p*. The deviation between observed and calculated values of librational frequencies is minimized when $1/\delta$ takes a maximum value.

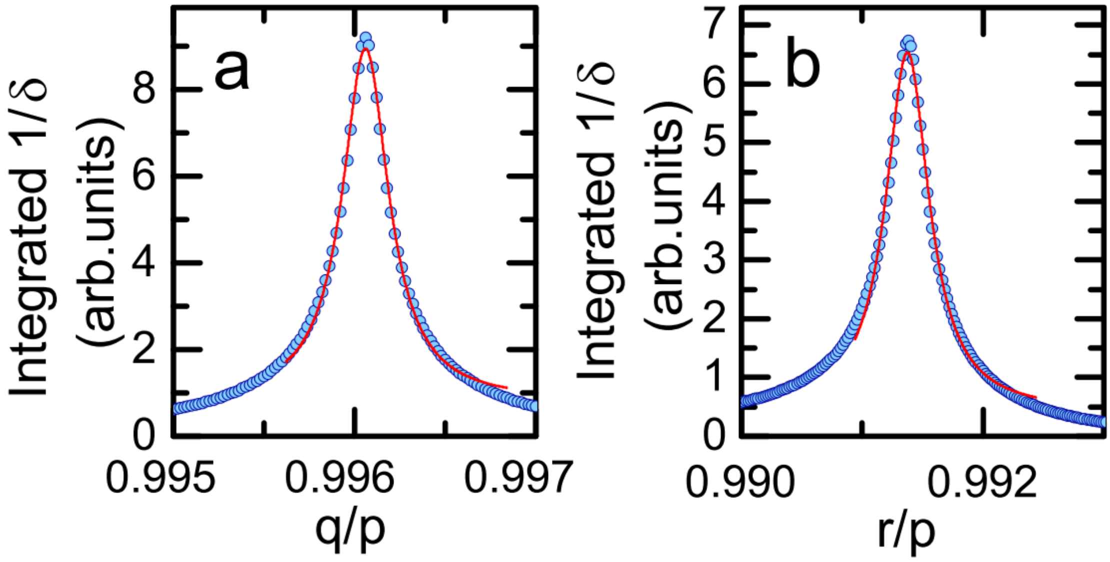

Supplementary Figure 5 $1/\delta$ integrated along one axis. **a,** Integrated along *r/p*. **b,** Integrated along *q/p*. The solid lines are fits with Lorentzian functions. From the fits on the profiles, we determine the magnitude of two radii with respect the longest radius to be *q/p* = 0.9961 and *r/p* = 0.9914 such that the deviation between observed and calculated frequencies is minimized.

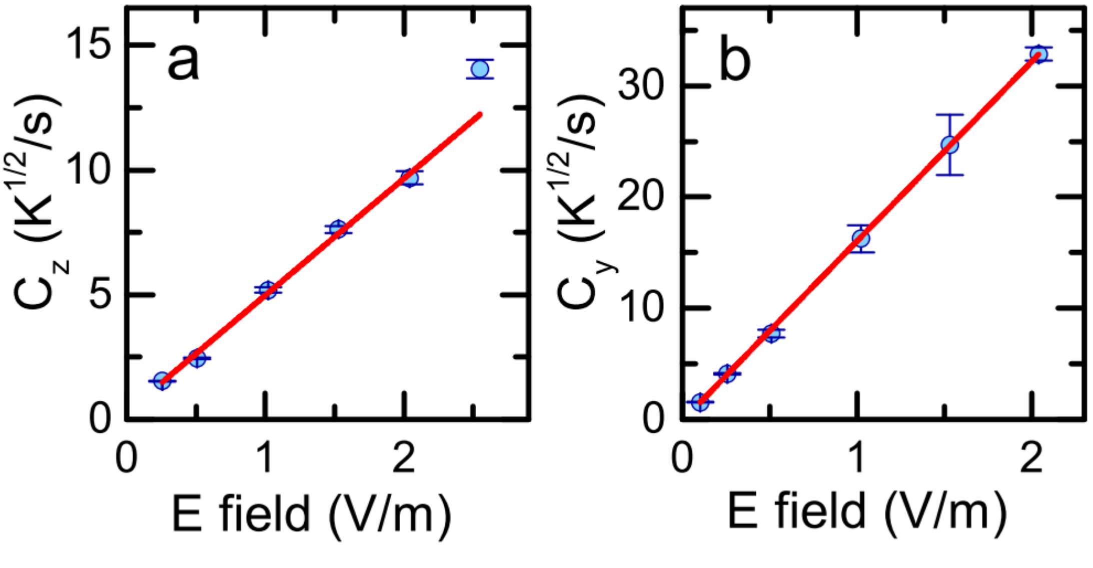

Supplementary Figure 6 Measured damping rates as a function of the magnitude of an applied electric field. **a,** For f_{lz}. **b,** For f_{ly}. The errors are statistical errors in determining the damping rates. The solid lines are linear fits. From the slopes of the plot, we determine the magnitude and the orientation of the electric dipole moment of the trapped nanoparticle.

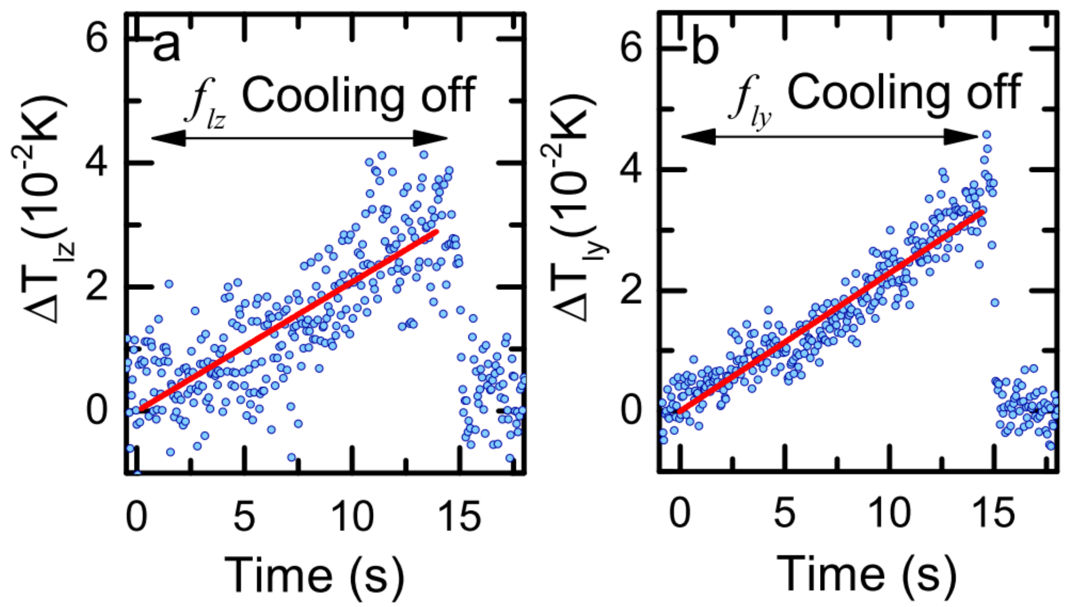

Supplementary Figure 7 Measured time variation of temperatures of librational motions after feedback cooling is turned off. a, For T_{lz}. **b,** For T_{ly}. The solid lines are linear fits. The observed slow heating rate of around 2 mK/s is limited by background gas collisions and is nearly independent from orientations.

Supplementary Table 1 Estimation of systematic uncertainties in ***q/p*, *r/p***

Source	Uncertainty (10^2 ppm)
Fitting on δ	0.01
Mass density	1.0
Light intensity (fluctuation)	1.7
Refractive index	5.1
Higher order terms ignored in calculations	0.5
Light polarization	0.03
Radius measurement	5.7
Frequency measurement	1.3
Total	8.0

Frequency variation due to anharmonicity.

Here, we provide the detailed derivation of Eq. (1). The equation of librational motions around the i axis is given by

$$I_i \frac{d^2\psi_i}{dt^2} = -\frac{1}{2} I_i (2\pi f_{li0})^2 \sin 2\psi_i$$

Given that librational motions are observed with narrow spectral width, we can safely assume $\psi_i(t) = A(t)\sin(2\pi f_{li} t)$with$\dot{A} \ll 2\pi A f_{li}$. Then the librational frequencies with a finite amplitude A is given by

$$f_{li} = f_{li0}\sqrt{\frac{\sin(2A\sin(2\pi f_{li}t))}{2A\sin(2\pi f_{li}t)}} \quad \text{(SI\_1)}$$

When the above equation is expanded in terms of $2A\sin(2\pi f_{li}t)$ as the Taylor series, the following equation is obtained.

$$f_{li} = f_{li0}\sqrt{\frac{\mathrm{x} - \frac{\mathrm{x}^3}{3!} + \frac{x^5}{5!} - \frac{x^7}{7!} + O(x^9)}{x}}$$

where $x = 2A\sin(2\pi f_{li}t)$. Because the above equation is an even function for x, it can be written as a function of $\sin^2(2\pi f_{li}t)$. Considering that the time average of $\sin^2(2\pi f_{li}t)$ is $1/2$, we substitute $1/\sqrt{2}$ for $\sin(2\pi f_{li}t)$ in the Eq. (SI_1) and obtain the following equation as the time average.

$$f_{li} = f_{li0}\sqrt{\frac{\sin\sqrt{2}A}{\sqrt{2}A}}$$

The temperatures of librational motions are given by the sum of the potential energy and the kinetic energy:

$$k_B T_{li} = \frac{1}{2} I_i (2\pi f_{li0})^2 \left(\frac{A}{2\sqrt{2}}\sin\sqrt{2}A + \sin^2\frac{A}{\sqrt{2}}\right)$$

which can be approximated as

$$k_B T_{li} \simeq \frac{1}{4} I_i (2\pi f_{li0})^2 \left(2A^2 - \frac{1}{2}A^4\right)$$

By solving this equation, we can replace relate A and T_{li} as

$$A \simeq \sqrt{2 - \sqrt{4 - \frac{2k_B T_{li}}{I_i(\pi f_{li0})^2}}}$$

Thus we obtain

$$f_{li} = f_{li0}\sqrt{\frac{\sin\sqrt{2}\beta}{\sqrt{2}\beta}}$$

With $\beta = \sqrt{2 - \sqrt{4 - 2k_B T_{li}/I_i(\pi f_{li0})^2}}$.

Derivation of the librational frequencies.

We calculate the potential energy of a dielectric ellipsoid in a Gaussian standing wave with the Rayleigh Gans

approximation. We assume that the center of mass (CoM) of the ellipsoid is fixed at the origin by feedback cooling and the orientation of the ellipsoid is described in terms of the Euler angles $(\alpha_E, \beta_E, \gamma_E)$ in the z-y'-z'' convention[1]. α represents the rotation of the laboratory frame around the z-axis ((x, y, z) to (x', y', z)). β represents the rotation around the y'-axis ((x', y', z) to (x'', y', z'')). γ represents the rotation around the z''-axis ((x'', y', z'') to (x''', y''', z'')). When $\alpha_E = \beta_E = \gamma_E = 0$, the radii of the ellipsoid are p along the x-axis, q along the y-axis, and r along the z-axis (Fig. 1).

To describe our experimental system, the incident beam is assumed to have elliptical beam waists w_x and w_y, while the retro-reflected beam has a symmetric beam waist w_0. Each wave has the wave number $k = \pm 2\pi/\lambda$, where λ is the wavelength, and is polarized in the x direction. Near the focal point, the electric field of the incoming beam E^i and reflected beam E^r takes the following form.

$$\overrightarrow{E^i} = E_0^i \exp\left[-\left(\frac{x^2}{w_x^2} + \frac{y^2}{w_y^2}\right) + ikz\right]\overrightarrow{n_x} \qquad \text{(SI\_2)}$$

$$\overrightarrow{E^r} = E_0^r \exp\left[-\left(\frac{x^2}{w_0^2} + \frac{y^2}{w_0^2}\right) - ikz\right]\overrightarrow{n_x} \qquad \text{(SI\_3)}$$

Then, the electric field of Gaussian standing wave is described as $\vec{E} = \overrightarrow{E^i} + \overrightarrow{E^r}$.

The potential energy due to the interaction between the light and the dielectric in the Rayleigh-Gans approximation[1] is described as following.

$$U = -\frac{1}{4}\int \vec{P}(r)\cdot\vec{E}(r)d^3r \ . \qquad \text{(SI\_4)}$$

where the integral region is the volume of dielectric, $\vec{P}(r) = \epsilon_0 \overleftrightarrow{R^T}\chi_0\overleftrightarrow{R}\vec{E}(r)$ is the polarization vector with ϵ_0, χ_0, and $\overleftrightarrow{R}$ being the permittivity, the diagonal susceptibility matrix in the nanoparticle frame, and the rotation matrix, respectively. χ_0 and $\overleftrightarrow{R}$ are given by

$$\overleftrightarrow{R} = \begin{pmatrix} c\beta c\alpha c\gamma - s\alpha s\gamma & c\beta s\alpha c\gamma + c\alpha s\gamma & -s\beta c\gamma \\ -c\beta c\alpha s\gamma - s\alpha c\gamma & -c\beta s\alpha s\gamma + c\alpha c\gamma & s\beta s\gamma \\ s\beta c\alpha & s\beta s\alpha & c\beta \end{pmatrix}, \quad \chi_0 = \begin{pmatrix} \chi_x & 0 & 0 \\ 0 & \chi_y & 0 \\ 0 & 0 & \chi_z \end{pmatrix}$$

where c=cos, s=sin, and χ_i $(i = x, y, z)$ is the susceptibility of the nanoparticle in the i direction of nanoparticle frame. χ_x is calculated following:

$$\chi_x = \frac{1}{\left(L_x + \frac{1}{n^2 - 1}\right)}, \qquad L_x = \int_0^\infty \frac{pqr \ \mathrm{d}s}{2(s+p^2)^{\frac{3}{2}}(s+q^2)^{\frac{1}{2}}(s+r^2)^{\frac{1}{2}}}$$

where n is the refractive index of the nanoparticle. Similarly, the χ_y and χ_z can be calculated. Substituting Eq. (SI_2) and Eq. (SI_3) for Eq. (SI_4), we obtain

$$U = -\frac{1}{4}\epsilon_0\chi' \int {E_0^i}^2 \exp\left[-2\left(\frac{x^2}{w_x^2} + \frac{y^2}{w_y^2}\right)\right] + {E_0^r}^2 \exp\left[-2\left(\frac{x^2}{w_0^2} + \frac{y^2}{w_0^2}\right)\right]$$
$$+ 2E_0^i E_0^r \exp\left[-\left(\frac{x^2}{w_x^2} + \frac{y^2}{w_y^2} + \frac{x^2}{w_0^2} + \frac{y^2}{w_0^2}\right)\right] 2\cos(2kz)\, d^3r$$

where $\chi' = (c\beta c\alpha c\gamma - s\alpha s\gamma)^2\chi_x + (-c\beta c\alpha s\gamma - s\alpha c\gamma)^2\chi_y + (s\beta c\alpha)^2\chi_z$. Since $x^2 \ll w_i^2 (i = x, y, 0)$, $y^2 \ll w_i^2$, $z^2k^2 \ll 1$ in the integral region, we ignore the fourth and subsequent orders of w_i^{-1} and k. The potential energy is then approximated as follows:

$$U \simeq -\frac{1}{4}\epsilon_0\chi' \int \Bigg[{E_0^i}^2 \left(1 - 2\left(\frac{x^2}{w_x^2} + \frac{y^2}{w_y^2}\right)\right) + {E_0^r}^2 \left(1 - 2\left(\frac{x^2}{w_0^2} + \frac{y^2}{w_0^2}\right)\right)$$
$$+ 4E_0^i E_0^r \left(1 - \left(\frac{x^2}{w_x^2} + \frac{y^2}{w_y^2} + \frac{x^2}{w_0^2} + \frac{y^2}{w_0^2}\right)\right)(1 - 2k^2z^2) \Bigg] d^3r$$

Here, we assume that the rotational motion is cooled to sufficiently small angular deviations and that each angle can be treated independently. Then we can expand the potential energy in terms of w_i^{-1} and k. Because of $w_i^{-1} < k$, we leave terms with up to $O(k^4)$. For sufficiently small angle deviations of ψ_x, ψ_y, and ψ_z, we can extract only the harmonic terms with respect to the oscillation angles ψ_x, ψ_y, and ψ_z. The potential energy is then decomposed to three terms with $O(\psi_x^2)$, $O(\psi_y^2)$, and $O(\psi_z^2)$, respectively, as follows:

$$U = U_{lx}^{(2)} + U_{ly}^{(2)} + U_{lz}^{(2)}$$

$$U_{lx}^{(2)} = V\epsilon_0 {E_0^i}^2 \frac{2}{5}(q^2 - r^2) \Big(-h_2$$
$$+ g\Big[2k^2(1 - \frac{2}{7}r^2k^2) - \frac{2}{7}(1 - \frac{2}{9}r^2k^2)p^2k^2l_1 - \Big\{1 + \frac{6}{7}(q^2 - r^2)k^2 + \frac{2}{63}(5r^2 - 6q^2)r^2k^4\Big\} l_2\Big]\Big) \chi_x \psi_x^2$$

$$U_{ly}^{(2)} = V \ \epsilon_0 {E_0^i}^2 \Big[\frac{2}{5}(p - r^2) \Big(-h_1$$
$$+ g\Big[2k^2(1 - \frac{2}{7}r^2k^2) - \Big\{1 - \frac{6}{7}(r^2 - p^2)k^2 + \frac{2}{63}(5r^2 - 6p^2)r^2k^4\Big\} l_1 - \frac{2}{7}(q^2k^2 - \frac{2}{9}q^2r^2k^4)l_2\Big]\Big) \chi_x$$
$$+ \Big(-(1 + g)^2$$
$$+ \frac{2}{5}\Big[(p^2h_1 + q^2h_2) + g\Big\{2r^2k^2(1 - \frac{1}{7}r^2k^2) + (p^2l_1 + q^2l_2)(1 - \frac{2}{7}k^2r^2 + \frac{2}{63}k^4r^4)\Big\}\Big]\Big) (-\chi_x$$
$$+ \chi_z)\Big] \psi_y^2$$

$$U_{lz}^{(2)} = V \ \epsilon_0 {E_0^i}^2 \Big(\frac{2}{5}(p^2 - q^2)(h_2 - h_1)\Big\{1 + g(1 - \frac{2}{7}r^2k^2 + \frac{2}{63}r^4k^4)\Big\} \chi_x$$
$$+ \Big[-(1 + g^2) + \frac{2}{5}(p^2h_1 + q^2h_2)$$
$$+ 2g\Big\{\frac{1}{5}(1 - \frac{2}{7}r^2k^2 + \frac{2}{63}r^4k^4)(p^2l_1 + q^2l_2) - (1 - \frac{2}{5}r^2k^2 + \frac{2}{35}r^4k^4)\Big\}\Big] (-\chi_x + \chi_y)\Big) \psi_z^2$$

where $g = E_0^r/E_0^i$ $h_1 = \frac{1}{w_x^2} + g^2\frac{1}{w_0^2}$, $h_2 = \frac{1}{w_y^2} + g^2\frac{1}{w_0^2}$, $l_1 = \frac{1}{w_x^2} + \frac{1}{w_0^2}$, $l_2 = \frac{1}{w_y^2} + \frac{1}{w_0^2}$. Considering that $U_{li}^{(2)} = I_i(2\pi f_{li})^2\psi_i^2$, we derive librational frequencies from these expressions and use them for the analysis of our data.

By ignoring higher order terms of $O(k^4)$, $O(w_i^{-4})$, $O(k^2w_i^{-2})$, and above, we can further simplify the potential energies as follows:

$$U_{lx}^{(2)} = \left[\frac{m}{10}(2\pi)^2(f_{tz}^2 - f_{ty}^2)(q^2 - r^2)\right]\psi_x^2$$

$$U_{ly}^{(2)} = \left[U_t\frac{|\alpha - \alpha_z|}{\alpha_x} + \left(\frac{m}{10}(2\pi)^2(f_{tx}^2 - f_{tz}^2)(r^2 - p^2)\right)\right]\psi_y^2$$

$$U_{lz}^{(2)} = \left[U_t\frac{|\alpha_x - \alpha_y|}{\alpha_x} + \left(\frac{m}{10}(2\pi)^2(f_{ty}^2 - f_{tx}^2)(p^2 - q^2)\right)\right]\psi_z^2$$

where $V = \frac{4}{3}\pi pqr$ is the volume of the ellipsoid, $\alpha_i = \epsilon_0 V \chi_i$ is the polarizability of the nanoparticle in the i direction of particle frame. $f_{tx} = \sqrt{\frac{{E_0^i}^2}{m\pi^2}\left(\frac{1}{w_x^2} + g^2\frac{1}{w_0^2} + g\left(\frac{1}{w_x^2} + \frac{1}{w_0^2}\right)\right)\alpha_x}$, $f_{ty} = \sqrt{\frac{{E_0^i}^2}{m\pi^2}\left(\frac{1}{w_y^2} + g^2\frac{1}{w_0^2} + g\left(\frac{1}{w_y^2} + \frac{1}{w_0^2}\right)\right)\alpha_x}$,

and $f_{tz} = \sqrt{\frac{2{E_0^i}^2}{m\pi^2} g k^2 \alpha_x}$ denote the CoM oscillation frequencies.

Thus, we obtain the approximate representations for three librational frequencies:

$$f_{lx} = \sqrt{\frac{(f_{tz}^2 - f_{ty}^2)(q^2 - r^2)}{q^2 + r^2}}$$

$$f_{ly} = \sqrt{\frac{(f_{tx}^2 - f_{tz}^2)(r^2 - p^2)}{p^2 + r^2} + \frac{10U_t(\alpha_x - \alpha_z)}{4\pi^2 m(p^2 + r^2)\alpha_x}}$$

$$f_{lz} = \sqrt{\frac{(f_{ty}^2 - f_{tx}^2)(p^2 - q^2)}{p^2 + q^2} + \frac{10U_t(\alpha_x - \alpha_y)}{4\pi^2 m(p^2 + q^2)\alpha_x}}$$

Time evolution of librational motion in the presence of feedback torque.

We provide the detailed derivation of Eq. (2). The equation of librational motions around the i axis in the presence of the feedback torque is given by

$$I_i \frac{d^2\psi_i}{dt^2} + \frac{1}{2} I_i (2\pi f_{li0})^2 \sin 2\psi_i = -I_i\gamma_i \frac{d\psi_i}{dt} \quad \text{(SI\_5)}$$

The right-hand side of this equation is about the feedback torque. When the relative phase between the feedback signal and PD is chosen to be 90 degrees, the feedback torque is written as following too:

$$N_f = dE_0\eta_i \sin\left(2\pi f_{li0} t - \frac{\pi}{2}\right)$$

Then, the damping rate γ_i is written as

$$\gamma_i = \frac{dE_0\eta_i}{2\pi I_i A f_{li0}} \quad \text{(SI\_6)}$$

From Eq. (SI_5), we obtain the following equation:

$$\frac{d}{dt}\left(\frac{1}{2} I_i \left(\frac{d\psi_i}{dt}\right)^2 + \frac{1}{2} I_i (2\pi f_{li0})^2 \sin^2\psi_i\right) = -I_i\gamma_i\left(\frac{d\psi_i}{dt}\right)^2$$

The left-hand side of this equation is time derivative of the sum of the potential energy and the kinetic energy, and is related to the temperature of librational motions. Since the anharmonic term makes no significant difference to the results, we can approximate the equation as

$$\frac{d}{dt}\left(\frac{1}{2} I_i \left(\frac{d\psi_i}{dt}\right)^2 + \frac{1}{2} I_i (2\pi f_{li0})^2 \psi_i^2\right) = -I_i\gamma_i\left(\frac{d\psi_i}{dt}\right)^2$$

Then, we can assume that $\psi_i(t) = A(t)\sin(2\pi f_{li0} t)$. Under the assumption that the time variations of the amplitude of ψ_i are slow, implying $\dot{A} \ll 2\pi A f_{li0}$, we write down the equation following:

$$\frac{d}{dt}\left(\frac{1}{2} I_i (2\pi f_{li0})^2 A^2\right) = -\frac{1}{2} I_i\gamma_i (2\pi f_{li0})^2 A^2 \quad \text{(SI\_7)}$$

Note that we use effective value of $\cos^2(2\pi f_{li0}t) = 1/2$ to obtain the time evolution of A. From the left-hand side of this equation, we obtain the relationship between A and T_{li}:

$$A = \frac{1}{2\pi f_{li0}}\sqrt{\frac{2k_B T_{li}}{I_i}} \tag{SI\_8}$$

Then we arrive at the equation for T_i from Eq. (SI_6) to Eq. (SI_8):

$$\frac{d}{dt}T_{li} = -\frac{dE_0\eta_i}{\sqrt{2I_i k_B}}\sqrt{T_{li}}$$

whose solution is given by Eq. (2).

Temperature of surrounding gasses.

It is known that the absorption of light increases the internal temperature of levitated nanoparticles. Particularly in regions of low pressures, the surrounding gas temperature increases due to the increase in the internal temperature of nanoparticles, so that librational motions are thermalized at temperatures higher than room temperature. We calculate the temperature of surrounding gasses T_{bath} from the internal temperature at the pressure of 6.5×10^{-7} Pa.

The internal temperature of a levitated particle depends on the absorption of the trap beam, absorption or radiation blackbody energy, and cooling due to collisions with background gases. We calculate the internal temperature following the Ref.[2], and the temperature of surrounding gases T_{bath} following the Ref.[3]. As a result, the temperature of surrounding gases T_{bath} is about 340 K in our setup.

We assume following situation, and use following parameters to calculate the T_{bath}:
Intensity of the trap beam at the trap point $I_0 = 13.1$ MW/cm^2, energy accommodation coefficient[3] $\alpha_c = 0.65$, specific heat ratio of a diatomic gas $\gamma = 7/5$, room temperature $T_{air} = 295$ K, the surrounding gas is N_2, the refractive index of silica[4] $n = 1.45 + (2.5 \times 10^{-9})i$, the value about blackbody radiation[4] $\mathrm{Im}\big((\epsilon_{bb} - 1)/(\epsilon_{bb} + 2)\big) = 0.1$, with ϵ_{bb} is the permittivity when the sphere has a relatively constant and temperature-independent permittivity across the blackbody radiation spectrum.

Correlation between each librational peaks

We observed the correlation between each librational peaks in terms of the oscillation frequency.
This measurement was performed with a different particle from the one used in the main text.
Supplementary Figure 8 shows the results of measuring f_{lx} and f_{lz} while changing the f_{ly}. The electrical feedback control was used to change f_{ly}. This figure shows that there is a positive correlation between f_{lx} and f_{ly}, and f_{lz} and f_{ly}.

Dependence of librational frequencies on laser power

The librational frequencies are measured while changing the trap laser power (Supplementary Figure 9).

This measurement was performed with a different particle from the one used in the main text.
We used following equation for fitting.

$$f_{li} = A\sqrt{P} \tag{SI\_9}$$

where P is the power of the laser and A is a coefficient of fitting. From this figure, we find that f_{lx}, f_{ly} and f_{lz} are proportional to the square-root of laser power.

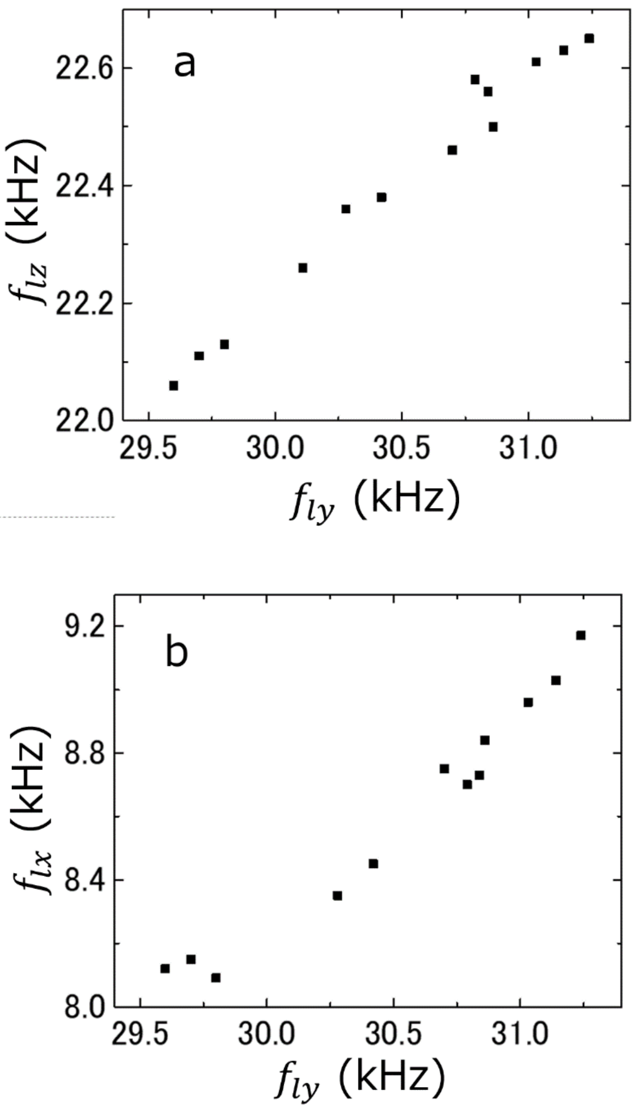

Supplementary Figure 8 Correlation between each librational frequencies. a, Measured frequency f_{lz} with respect to the f_{ly}. **b,** Measured frequency f_{lx} with respect to the f_{ly}.

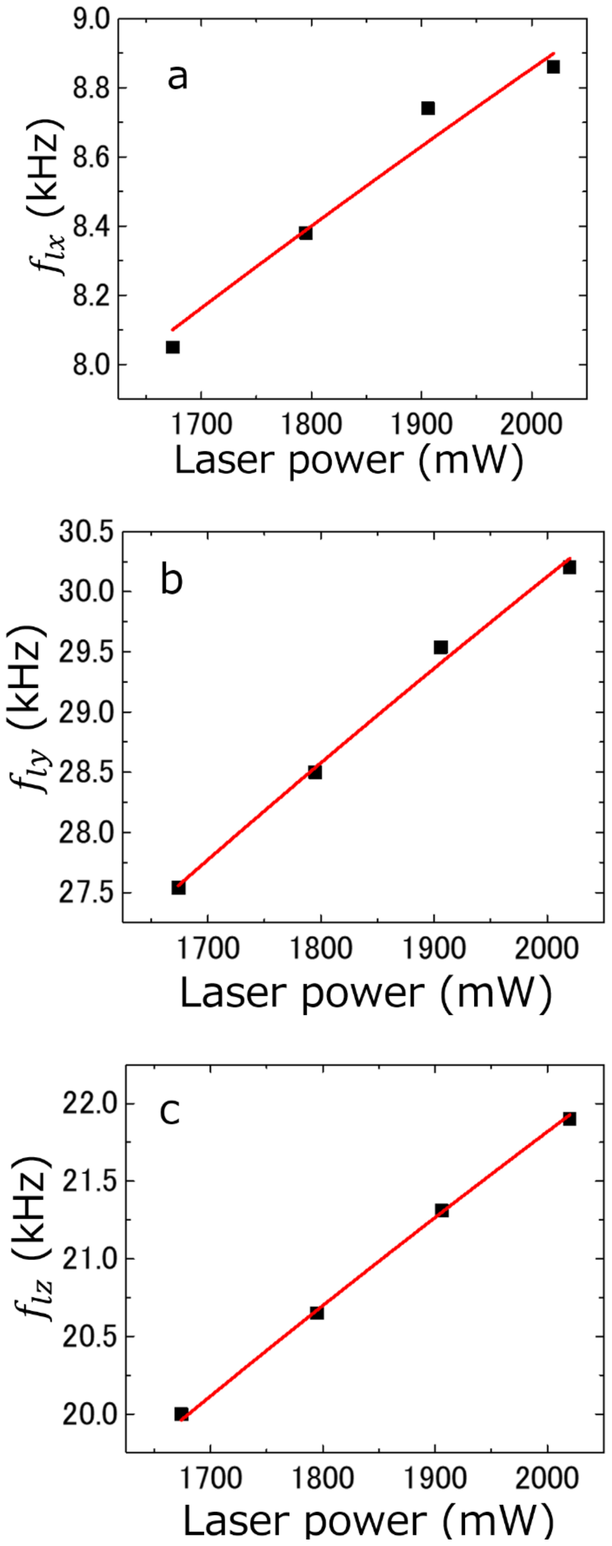

Supplementary Figure 9 Measured frequencies variation as a function of laser power. The solid line is the fitting of data with Eq.(SI 9). **a,** Measured frequency f_{lx} with respect to laser power. **b,** Measured frequency f_{ly} with respect to laser power. **c,** Measured frequency f_{lz} with respect to laser power.

Measurements of the refractive index of the SiO_2 nanoparticles.

We measure the refractive index of SiO_2 nanoparticles used in the present study at the wavelength of 1550 nm in the following manner. First, we measure the transmissivity of 0.5 cc of the refractive index liquid (Cargille Laboratories) filled in a glass cell without adding the SiO_2 sample. Then, we add 60 mg of the sample to the liquid and ultrasonicate the mixture for 10 minutes. The nanoparticles are heavier than the liquid and sink near the bottom of the glass cell. We stir the liquid and measure the transmissivity of the mixture right after stirring. By comparing the transmissivity with and without the sample, we obtain the normalized transmissivity as a function of the refractive index of the liquid. The same procedure is repeated for 12 indices between 1.406 and 1.464 (Supplementary Figure 10). The values of the refractive index of the liquid at 1550 nm are provided by the manufacturer. From the fit on the measured data, we determine the refractive index of our sample to be 1.43025(32). Although the statistical error of the measurement is fairly small, there might be a particle-to-particle variation in the refractive index. Therefore, as an upperbound, we take the HWHM of the measured peak as a systematic error of the refractive index, which corresponds to 0.76%.

The obtained value is slightly smaller than the value of 1.45 known for bulk SiO_2 at 1550 nm. For nanoparticles, only a few measurements at visible wavelengths are reported. When we assume that the variation of the refractive index of nanoparticles with respect to the wavelength is the same as that of bulk SiO_2, we obtain estimated values of 1.456 from Ref. 5 and 1.397 from Ref.6. Thus, the value obtained in the present work shows a reasonable agreement with previous studies.

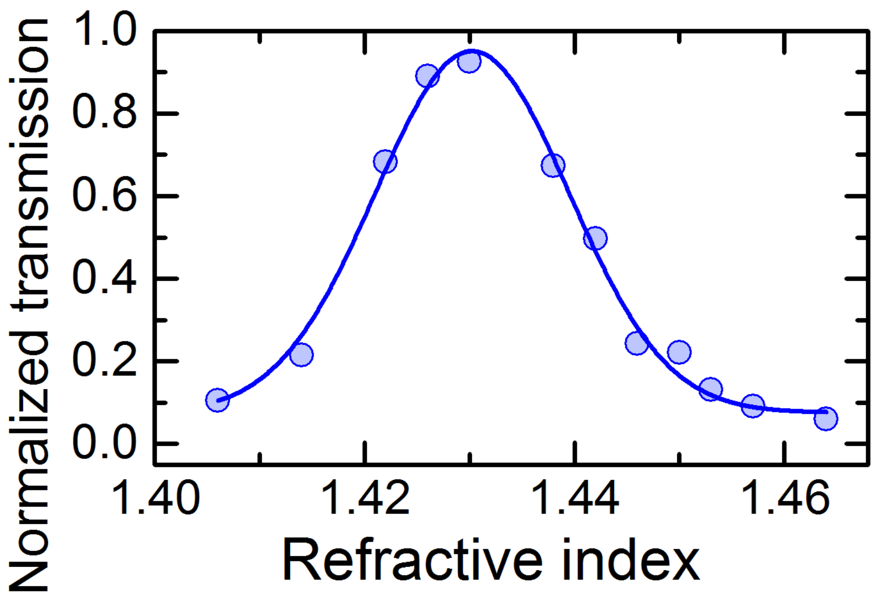

Supplementary Figure 10 Normalized transmission as a function of the refractive index of the liquid. The solid line is a fit on the measured data with a Gaussian function.

Supplementary References

1. T. Seberson and F. Robicheaux, *Stability and dynamics of optically levitated dielectric disks in a Gaussian standing wave beyond the harmonic approximation*, Phys. Rev. Research **2**, 033437 (2020)
2. D. E. Chang, C. A. Regal, S. B. Papp, D. J. Wilson, J. Ye, O. Painter, H. J. Kimble, and P. Zoller, *Cavity opto-mechanics using an optically levitated nanosphere*, Proc. Natl. Acad. Sci. U.S.A. **107**, 1005 (2010).
3. E. Hebestreit, R. Reimann, M. Frimmer, and L. Novotny, *Measuring the internal temperature of a levitated nanoparticle in high vacuum*, Phys. Rev. A **97**, 043803 (2018).
4. J. Millen *et. al*. *Optomechanics with levitated particles*, Rep. Prog. Phys. **83** 026401 (2020).
5. B. N. Khlebtsov, *et. al.*, *Determination of the Size, Concentration, and Refractive Index of Silica Nanoparticles from Turbidity Spectra*, Langmuir **24**, 8964-8970 (2008).
6. S. Kimoto, *et. al.*, *Characterization of nanosized silica size standards*, Aerosol Sci. Technol. **51**, 936-945 (2017).
